# Predictors of intracranial hemorrhage in adult patients on extracorporeal membrane oxygenation: an observational cohort study

**DOI:** 10.1186/s40560-017-0223-2

**Published:** 2017-05-22

**Authors:** Alexander Fletcher Sandersjöö, Jiri Bartek, Eric Peter Thelin, Anders Eriksson, Adrian Elmi-Terander, Mikael Broman, Bo-Michael Bellander

**Affiliations:** 1 Department of Neurosurgery, Karolinska University Hospital, Stockholm, Sweden; 2 Department of Clinical Neuroscience, Karolinska Institutet, Stockholm, Sweden; 3 Department of Neurosurgery, Copenhagen University Hospital Rigshospitalet, Copenhagen, Denmark; 4 Division of Neurosurgery, Department of Clinical Neurosciences, Cambridge Biomedical Campus, University of Cambridge, Cambridge, UK; 5 ECMO Center Karolinska, Department of Pediatric Perioperative Medicine and Intensive Care, Karolinska University Hospital, Stockholm, Sweden; 6 Department of Physiology and Pharmacology, Karolinska Institutet, Stockholm, Sweden

**Keywords:** Intracranial hemorrhage, Extracorporeal membrane oxygenation, Adults, Predictors, Risk factors

## Abstract

**Background:**

Intracranial hemorrhage (ICH) is a recognized complication of adults treated with extracorporeal membrane oxygenation (ECMO) and is associated with increased morbidity and mortality. However, the predictors of ICH in this patient category are poorly understood. The purpose of this study was to identify predictors of ICH in ECMO-treated adult patients.

**Methods:**

We conducted a retrospective review of adult patients (≥18 years) treated with ECMO at the Karolinska University Hospital (Stockholm, Sweden) between September 2005 and June 2016, excluding patients with ICH upon admission or those who were treated with ECMO for less than 12 h. In a comparative analysis, the primary end-points were the difference in baseline characteristics and predictors of hemorrhage occurrence (ICH vs. non-ICH cohorts). The secondary end-point was difference in mortality between groups. Paired testing and uni- and multivariate regression models were applied.

**Results:**

Two hundred and fifty-three patients were included, of which 54 (21%) experienced an ICH during ECMO treatment. The mortality for patients with ICH was 81% at 1 month and 85% at 6 months, respectively, compared to 28 and 33% in patients who did not develop ICH. When comparing ICH vs. non-ICH cohorts, pre-admission antithrombotic therapy (*p* = 0.018), high pre-cannulation Sepsis-related Organ Failure Assessment (SOFA) coagulation score (*p* = 0.015), low platelet count (*p* < 0.001), and spontaneous extracranial hemorrhage (*p* = 0.045) were predictors of ICH. In a multivariate regression model predicting ICH, pre-admission antithrombotic therapy and low platelet count demonstrated independent risk association. When comparing the temporal trajectories for coagulation variables in the days leading up to the detection of an ICH, plasma antithrombin significantly increased per patient over time (*p* = 0.014). No other temporal trajectories were found.

**Conclusions:**

ICH in adult ECMO patients is associated with a high mortality rate and independently associated with pre-admission antithrombotic therapy and low platelet count, thus highlighting important areas of potential treatment strategies to prevent ICH development.

**Electronic supplementary material:**

The online version of this article (doi:10.1186/s40560-017-0223-2) contains supplementary material, which is available to authorized users.

## Background

Extracorporeal membrane oxygenation (ECMO) for respiratory and circulatory support—frequently used in pediatric intensive care [1]—is increasingly being employed in adults [2–4]. In addition to the critical condition of the patients treated, there is significant morbidity associated with the ECMO treatment itself [5]. A frequent complication is bleeding, a result of the systemic effects of cardiopulmonary bypass, causing platelet dysfunction, platelet consumption, and hemodilution of clotting factors, in combination with the anticoagulation and antiplatelet therapy administered to reduce the risk of circuit clotting [6]. In all probabilities, intracranial hemorrhage is the most devastating bleeding complication that can occur (ICH) [7] and has previously been associated with increased mortality in adults receiving ECMO treatment [8–10]. Despite this, the predictors of ICH in adult patients treated with ECMO are poorly understood.

So far, studies investigating the predictors of ICH in ECMO patients have focused on the pediatric population [11, 12] with only three studies looking into possible predictors in adults. In these studies, female sex, thrombocytopenia, use of heparin, dialysis, creatinine >2,6 mg/dl (230 μmol/L), duration of ECMO treatment, increased activated clotting time (ACT), spontaneous extracranial hemorrhage, renal failure upon intensive care unit (ICU) admission as well as rapid PaO_2_ increase and PaCO_2_ decrease upon ECMO initiation have been identified as predictors of ICH [13–15]. Generally, these studies have been constrained by the limited number of patients included, making statistical modeling difficult. In addition, only one of them included patients treated with both venoarterial (VA) and venovenous (VV) ECMO.

In this largest retrospective observational cohort study to date, we explored possible predictors of ICH in ECMO-treated adult patients.

## Methods

### Patients

All adult patients (≥18 years) treated with VA or VV ECMO at the Karolinska University Hospital, Stockholm, Sweden, between September 2005 and June 2016 were eligible for inclusion. Patients with intracranial hemorrhage upon admission were excluded. To reduce the influence of precipitating events, patients were required to have been on ECMO support for at least 12 h prior to decannulation or the development of an ICH. Medical records, including clinical notes, laboratory analysis, monitoring reports, and brain imaging data were collected from patient charts. The outcome variable was the presence of an ICH on a computed tomography (CT) scan.

### Variables

#### Pre-cannulation data

Medical history and clinical charts were retrospectively reviewed, and the following pre-cannulation data were collected: age, sex, comorbidities (including a calculation of the Charlson comorbidity index [16]), Sepsis-related Organ Failure Assessment (sometimes referred to as Sequential Organ Failure Assessment) (SOFA) scores [17], and an arterial blood gas analysis within 2 h before cannulation. Ongoing antithrombotic therapy prior to admission (defined in our study as both antiplatelet and anticoagulation therapy), as well as cardiopulmonary resuscitation at any point during hospitalization were also noted.

#### ECMO data

ECMO data included the indication for ECMO treatment, the ECMO mode employed (VA or VV ECMO), and whether conversion (shifting from one mode to the other) was necessary. Common complications that occurred during the ECMO treatment were also registered, including septic shock, dialysis, spontaneous extracranial hemorrhage, and administered blood products post-cannulation (plasma, platelets, and erythrocyte concentrate). We also noted the lowest recorded value during the ECMO treatment for platelet count, fibrinogen concentration, P_v_CO_2_ and axillary body temperature, as well as the highest recorded value for antithrombin concentration, international normalized ratio (INR), venous lactate concentration, venous pH, P_v_O_2_, and mean arterial blood pressure (MAP). By registering the highest/lowest values, as opposed to medians or means, we were able to catch temporary critical levels that could precipitate ICH development, for example, unexpected increases in MAP or acute decreases in platelet count. For patients that developed an ICH, variables were only recorded until the detection of the ICH, which in turn was determined according to the time the CT scan was performed. Registered follow-up data were 1-month and 6-month mortality. In addition, for patients with ICH, we registered hemorrhage location, hemorrhage volume (calculated by multiplying the hemorrhage length × width × height and dividing by two), and Fisher scale [18]. We also registered the daily mean platelet count, antithrombin concentration, fibrinogen concentration, INR and activated partial thromboplastin time (APTT) on the day the ICH was diagnosed as well as in the 4 days preceding diagnosis—an arbitrary time frame we believe would incorporate any clinically important information.

### Patient management during ECMO

Patients with potentially reversible acute respiratory and/or cardiac failure were considered for ECMO treatment. In acute respiratory failure, a ratio of arterial oxygen partial pressure to fraction of inspired oxygen (P_a_O_2_/F_i_O_2_ ratio) <80 mmHg (FiO_2_ 1.0) was required. Other criteria included peak inspiratory pressure >35 cmH_2_O (pressure control), prolonged refractory hypercarbia with acidosis (pH < 7.10), and Murray score >3 [19]. In acute cardiac failure, the following criteria increased the chance of acceptance for ECMO treatment: central venous oxygen saturation (S_cv_O_2_) <55%, cardiac index <2 L/min m^2^ [20], acidaemia, lactatemia, or vasoactive-inotropic score >45–50 μg/kg min^−1^ [21].

Anticoagulation was achieved by a continuous intravenous infusion of unfractionated heparin (UFH) targeting an APTT of 60–80 s, which was assessed three times daily. Hourly monitoring using arterial and/or venous blood gas analysis was performed (Radiometer, Copenhagen, Denmark), including a separate assessment for activated clotting time (ACT) (Hemochron Mini II, Helena Laboratories, Beaumont, TX, USA, or Hemochron Junior, Scandinavian Medical Partner, Gothenburg, Sweden) with a treatment target of 180–220 s. After arrival at our ICU, a tracheostomy was performed within 2 to 3 days, after which the patients’ sedation was reduced with the goal of keeping the patient awake with analgosedation. The bedside nurse and the physician in charge of the patient continuously monitored the central nervous system through serial neurological examinations. This included calculation of the Glasgow Coma Scale [22], response to verbal directives or pain, brainstem reflexes, eye opening, and pupil examination. When an unexpected neurological event occurred (e.g., seizures, mydriasis, anisocoria, delirium, confusion, motor function deficits, or failure to wake up following withdrawal of sedation), a cerebral CT scan was performed. Additional cerebral CT scans were also performed whenever the patient was referred for a thoracic or abdominal CT scan, even without any apparent clinical indication. A description of the ECMO pumps, oxygenators, ventilators, cannulas and patients monitoring systems used is included in Additional file 1.

### Statistical analysis

For descriptive purposes, continuous data are presented as medians (interquartile range) and categorical data as numbers (proportion). Mann-Whitney *U* test and chi-square test were used to compare continuous and categorical variables, respectively. A univariate regression analysis was then used to correlate factors indicating a significant trend (*p* < 0.1) between ICH and non-ICH cohorts (“lrm” function in R, “rms”-package) [23]. In the univariate model, un-imputed data were used. Nagelkerke’s pseudo-*R* ^2^ was used to illustrate the pseudo explained variance, where “0” does not explain and “1” fully explains the model. A multivariate model, bias-adjusted for multiple parameters, was performed to determine independent risk factors for ICH. In order to detect significant temporal trajectories in the coagulation variables of patients that developed an ICH, a paired *t* test was used. Moreover, a student’s *t* test was used to detect any statistically significant change in the coagulation variables between the day of ICH diagnosis vs. 4 days prior. The statistical significance level was set to *p* < 0.05. The statistical program R was used, utilizing the interface R-studio Version 1.0.136 [23].

## Results

During the study period, 311 adults were admitted for ECMO treatment. Of these, 24 were excluded from our study due to <12 h of ECMO treatment, four were excluded due to the presence of an ICH upon admission and 30 were excluded because their ECMO treatment was partially conducted at a different hospital. Two hundred and fifty-three patients were included in the study, 161 of whom were treated with VV ECMO and 92 treated with VA ECMO, while 42 required conversion at least once. Pulmonary indications were by far the most common reason for ECMO treatment (*n* = 224, 89%). The majority of patients (98.4%) received intravenous heparin infusion to prevent clotting. The patients that did not receive heparin infusion did so because they had ongoing bleeding complications.

### ICH events and patient outcome

Fifty-four patients (21%) developed an ICH during their ECMO treatment. Forty-one of these were intracerebral hemorrhages (76%), 12 were subarachnoid hemorrhages (22%), and one was a subdural hemorrhage (2%). The median hematoma volume for the intracerebral hemorrhages was 22.8 mL, and the median Fisher scale for the subarachnoid hemorrhages was grade 2 (Table 1). The median time to ICH development from ECMO initiation was 7 days (4.0–14.5) (Table 4). Compared to non-ICH cohorts, ICH development was strongly associated with increased 1-month mortality (81 vs. 28%, *p* < 0.001, pseudo-*R* ^2^ = 0.258) and 6-month mortality (85 vs. 33%, *p* < 0.001, pseudo-*R* ^2^ = 0.248). There was no significant difference in ICH occurrence between VV and VA ECMO patients (19 and 27%, respectively, *p* = 0.283).

**Table 1:** ICH characteristics

Variables	ICH cohort (*n* = 54)
Intracerebral hemorrhage	41 (76%)
Hematoma volume (mL)^a^	22.8 (6.24–56.7)
Subdural hemorrhage	1 (2%)
Hematoma volume (mL)^a^	25.0 (range N/A)
Subarachnoid hemorrhage	12 (22%)
Fisher grade	2 (2–4)

Values are expressed as median (interquartile range) or numbers (proportion)

*Abbreviation: ICH* intracranial hemorrhage

^a^Calculated by multiplying the length × width × height of the hemorrhage and dividing by two

### Predictors of ICH

When comparing ICH with non-ICH cohorts, increased risk of ICH was associated with the following: pre-admission antithrombotic therapy (*p* = 0.018), high pre-cannulation SOFA coagulation score (*p* = 0.015) (Table 2), low platelet count (*p* < 0.001) (Table 3), spontaneous extracranial hemorrhage (*p* = 0.045), amount of administered platelets (*p* = 0.004), and amount of administered erythrocyte concentrate (*p* = 0.014) (Table 4).

**Table 2:** ICH vs. non-ICH cohorts: demographics, pre-admission morbidity, pre-cannulation SOFA score, ECMO mode, and indication

Variable	ICH cohort (*n* = 54)	Non-ICH cohort (*n* = 199)	*p* value
Demographics			
Age (years)	51.5 (40–61)	50 (32–60)	0.158
Male sex	35 (65%)	127 (64%)	1.000
Pre-admission morbidity			
Hypertension	9 (17%)	36 (18%)	0.967
Insulin-dependent diabetes mellitus	5 (9%)	22 (11%)	0.896
Chronic renal disease	2 (4%)	1 (1%)	0.116
Antithrombotic therapy	7 (13%)	7 (4%)	*0.018*
Charlson comorbidity index	0 (0–1)	0 (0–1)	0.373
Pre-cannulation SOFA score			
Total	14 (10.8–16) (26 missing, 48%)	13 (10–15) (106 missing, 53%)	0.443
Respiration	4 (4–4) (25 missing, 46%)	4 (4–4) (95 missing, 48%)	0.340
Coagulation	2 (0–3) (26 missing, 48%)	1 (0–2) (100 missing, 50%)	*0.015*
Liver	1 (0–2) (26 missing, 48%)	1 (0–2) (102 missing, 51%)	0.960
Cardiovascular	4 (3–4) (25 missing, 46%)	4 (3–4) (95 missing, 48%)	0.742
Neurological	1 (0–2) (25 missing, 46%)	1 (0–2) (98 missing, 49%)	0.726
Renal	1.5 (1–4) (26 missing, 48%)	1 (0–4) (101 missing, 51%)	0.606
ECMO Indication			
Pulmonary	48 (89%)	176 (88%)	
Cardiac	2 (4%)	17 (9%)	
ECPR	4 (7%)	6 (3%)	
ECMO mode			
Venovenous	31 (57%)	130 (65%)	0.361
Venoarterial	23 (43%)	69 (35%)	0.361

Values are expressed as median (interquartile range) or numbers (proportion)

Italicized text in the *p* value column indicates a statistically significant correlation (*p* < 0.05)

Pulmonary indications included pneumonia (*n* = 102), sepsis (*n* = 78), respiratory failure (*n* = 18), ARDS (*n* = 17), trauma (*n* = 6), and drowning (*n* = 3). Cardiac indications included cardiogenic shock (*n* = 18) and pulmonary embolism (*n* = 1)

*Abbreviations: SOFA* Sepsis-related Organ Failure Assessment (also known as Severity Organ Failure Assessment), *ECMO* extracorporeal membrane oxygenation, *ICH* intracranial hemorrhage, *ECPR* extracorporeal cardiopulmonary resuscitation

**Table 3:** ICH vs. non-ICH cohorts: laboratory variables

Variable	ICH cohort (*n* = 54)	Non-ICH cohort (*n* = 199)	*p* value
Lab biochemistry			
Platelet count^a^ (×10^9^/mL)	31 (17–46)	48 (27–84)	<*0.001*
INR^b^	1.5 (1.2–1.8)	1.4 (1.2–1.6)	0.147
Antithrombin^b^ (kIU/L)	0.90 (0.78–1.06)	0.93 (0.82–1.09)	0.542
Fibrinogen^a^ (g/L)	2.8 (1.5–4.3)	2.7 (1.6–3.8)	0.814
v-Lactate^b^ (mmol/L)	4.7 (3.0–9.7) (4 missing, 7%)	3.7 (2.7–7.7) (22 missing, 11%)	0.174
Blood gas analysis			
Arterial^c^			
a-pH	7.26 (7.18–7.33) (16 missing, 30%)	7.25 (7.16–7.34) (51 missing, 26%)	0.703
P_a_CO_2_ (kPa)	6.90 (6.10–7.65) (17 missing, 31%)	7.27 (6.00–9.90) (50 missing, 25%)	0.481
P_a_O_2_ (kPa)	7.39 (6.36–8.81) (16 missing, 30%)	7.34 (6.17–9.00) (50 missing, 25%)	0.661
Venous			
v-pH^a^	7.22 (7.17–7.26) (4 missing, 7%)	7.23 (7.17–7.28) (22 missing, 11%)	0.184
P_v_CO_2_ (kPa)^b^	7.80 (7.38–8.95) (4 missing, 7%)	8.04 (7.25–9.00) (22 missing, 11%)	0.890
P_v_O_2_ (kPa)^a^	4.30 (3.73–4.80) (4 missing, 7%)	4.46 (4.00–5.00) (22 missing, 11%)	0.105

Values are expressed as median (interquartile range) or numbers (proportion)

Italicized text in the *p* value column indicates a statistically significant correlation (*p* < 0.05)

*Abbreviations: ICH* intracranial hemorrhage, *INR* international normalized ratio

^a^Lowest value during ECMO support and before ICH diagnosis (if applicable)

^b^Highest value during ECMO support and before ICH diagnosis (if applicable)

^c^Registered within 2 h before cannulation

**Table 4:** ICH vs. non-ICH cohorts: clinical variables

Variable	ICH cohort (n = 54)	Non-ICH cohort (n = 199)	*p* value
Administered blood products^a^			
Plasma (mL)	2804 (1434–5674) (3 missing, 6%)	1873 (690–4887) (22 missing, 11%)	0.061
Platelets (mL)	1239 (275–3265) (3 missing, 6%)	370 (0–2072) (22 missing, 11%)	*0.004*
Erythrocyte concentrate (mL)	3576 (2155–6848) (3 missing, 6%)	2471 (1027–6531) (22 missing, 11%)	*0.014*
Mean arterial pressure^b^ (mmHg)	99 (89–113) (1 missing, 2%)	100 (92–109) (16 missing, 8%)	0.791
Axillary temperature <36 °C^a^	12 (23%) (1 missing, 2%)	53 (29%) (16 missing, 8%)	0.463
Spontaneous extracranial hemorrhage^a^	32 (59%)	85 (43%)	*0.045*
CPR^c^	11 (20%)	34 (17%)	0.719
Septic shock^a^	24 (44%)	59 (30%)	0.059
Dialysis^a^	52 (96%)	171 (86%)	0.063
Converted	12 (22%)	30 (15%)	0.296
ECMO days at ICH diagnosis	7 (4.0–14.5)	–	–
Days on ECMO	12 (5–19)	7 (4.0–15)	0.057
1-month mortality	44 (81%)	52 (28%) (13 missing, 7%)	*<0.001*
6-month mortality	46 (85%)	61 (33%) (13 missing, 7%)	*<0.001*

Values are expressed as median (interquartile range) or numbers (proportion)

Italicized text in the *p* value column indicates a statistically significant correlation (*p* < 0.05)

*Abbreviations: ICH* intracranial hemorrhage, *CPR* cardiopulmonary resuscitation, *ECMO* extracorporeal membrane oxygenation

^a^During ECMO support and before ICH diagnosis (if applicable)

^b^Highest value during ECMO support and before ICH diagnosis (if applicable)

^c^During hospitalization and before ICH diagnosis (if applicable)

### Independent predictors of ICH

In the regression analysis, we included variables with *p* < 0.1. The pre-cannulation SOFA coagulation score was not included because the data was missing in 50% of patients (the parameter was not registered at our center prior to 2012), which would require a high degree of imputation and weaken any conclusions. The SOFA coagulation score is determined by the platelet count, where a lower platelet count yields a higher score [17]. As expected, a strong co-variance between the pre-cannulation SOFA coagulation score and low platelet count (pseudo-*R* ^2^ = 0.156, data not shown) was also found, further supporting the removal of this variable from the regression analysis. Furthermore, due to a high degree of treatment intensity bias, we also disregarded the variables concerning administered blood products in the regression analysis. The univariate regression analysis confirmed pre-admission antithrombotic therapy (*p* = 0.014), low platelet count (*p* < 0.001), and spontaneous extracranial hemorrhage (*p* = 0.031) as predictors of ICH. It also recognized septic shock (*p* = 0.043) and dialysis (*p* = 0.020) as predictors. In the multivariate analysis, pre-admission antithrombotic therapy (*p* = 0.011, *R* ^2^ = 0.037) and low platelet count (*p* = 0.035, *R* ^2^ = 0.074) demonstrated independent risk association (Table 5).

**Table 5:** ICH risk factors: uni- and multivariate analysis

Variable	Univariate *p* value	Nagelkerke’s pseudo *R* ^2^	Multivariate *p* value
Pre-admission antithrombotic therapy	*0.014*	0.036	*0.011*
Platelet count	*<0.001*	0.074	*0.035*
Septic shock	*0.043*	0.025	0.280
Dialysis	*0.020*	0.033	0.215
Spontaneous extracranial hemorrhage	*0.031*	0.028	0.221

Italicized text in the *p* value column indicates a statistically significant correlation (*p* < 0.05)

*Abbreviations: ICH* intracranial hemorrhage, *ECMO* extracorporeal membrane oxygenation

### Coagulation trajectories prior to ICH

**Fig. 1:**
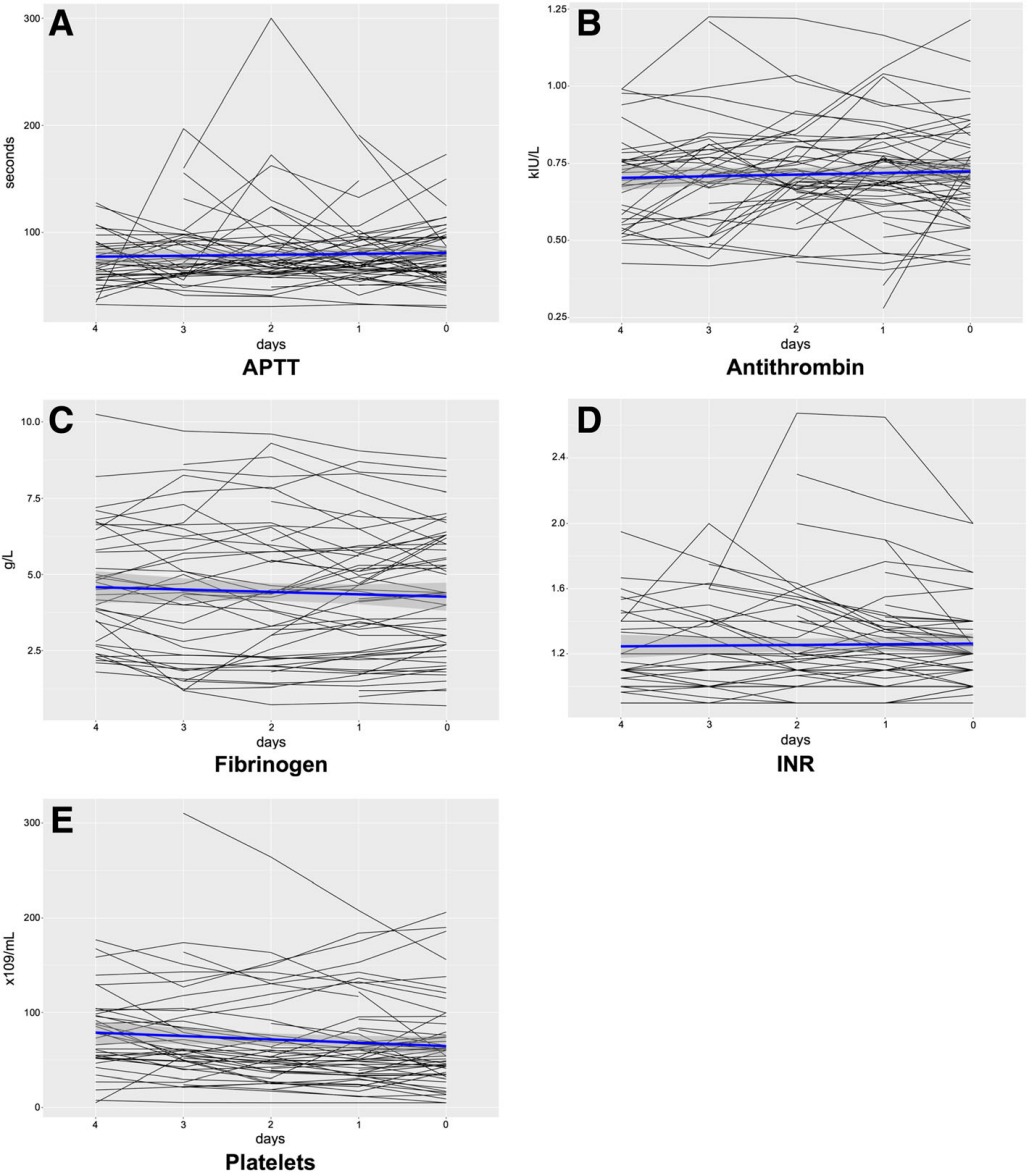
ICH cohort: temporal trajectories for coagulation variables prior to ICH diagnosis. Calculated using the daily means in the 4 days preceding the diagnosis of an ICH as well as on the day of ICH diagnosis. The *horizontal axis* indicates the amount of days prior to detection of an ICH, where “0” is the same day that the ICH was later detected. The *horizontal blue lines* represent the combined daily mean values for all patients, and the *gray area* represents the corresponding 95% confidence interval. *APTT* activated partial thromboplastin time, *INR* international normalized ratio

APTT, platelet count, INR, antithrombin, and fibrinogen concentration during the 4 days preceding the diagnosis of an ICH as well as on the day of ICH diagnosis were assessed. Figure 1 displays the daily means for each patient, and Table 6 demonstrates the combined mean values on the day of ICH diagnosis and 4 days prior to this. The results show that, on both an individual and group level, the APTT consistently stayed within the therapeutic range and there was no significant change in APTT preceding ICH diagnosis (*t* test *p* = 0.289, paired *t* test *p* = 0.254, Fig. 1, Table 6). In the paired *t* test, the only parameter that significantly changed per patient over time preceding ICH diagnosis was antithrombin (increasing levels) (*p* = 0.014). Apart from this, no other significant temporal trajectories were found and no significant difference on group level could be detected.

**Table 6:** ICH cohort: coagulation variables on the day of ICH diagnosis and 4 days prior

Variable	4 days prior to ICH diagnosis	Day of ICH diagnosis	*t* test *p* value	Paired *t* test *p* value
APTT (s)	69 (57–89)	78 (60–95)	0.289	0.254
Platelet count (×10^9^/mL)	76 (52–99)	54 (35–77)	0.156	0.263
Antithrombin (kIU/L)	0.70 (0.56–0.76)	0.71 (0.63–0.80)	0.374	*0.014*
Fibrinogen (g/L)	4.50 (2.80–6.13)	4.40 (2.70–6.00)	0.467	0.496
INR	1.13 (1.05–1.40)	1.20 (1.10–1.30)	0.816	0.054

Values are expressed as medians (interquartile range)

Italicized text in the *p* value column indicates a statistically significant correlation (*p* < 0.05)

*Abbreviations: APTT* activated partial thromboplastin time, *INR* international normalized ratio

## Discussion

In this observational cohort study, we sought to identify predictors of ICH in adult patients receiving ECMO treatment. Out of 253 patients, 54 (21%) developed an ICH. In patients that developed an ICH, the mortality was 81% at 1 month and 85% at 6 months, compared to 28 and 33%, respectively, in patients without an ICH. We identified (i) pre-admission antithrombotic therapy, (ii) high pre-cannulation SOFA coagulation score, (iii) low platelet count, (iv) septic shock, (v) dialysis, (vi) spontaneous extracranial hemorrhage, (vii) administered platelets, and (viii) administered erythrocyte concentrate as predictors of ICH development. Of these, pre-admission antithrombotic therapy and low platelet count were independent risk factors. Notably, this is the first time that pre-admission antithrombotic therapy, high pre-cannulation SOFA coagulation score, and septic shock have been identified as predictors of ICH. In addition, when comparing the temporal trajectories for coagulation variables in the days leading up to the detection of an ICH, there was a significant per patient increase in antithrombin concentration over time, while the means remained largely unchanged. To the best of our knowledge, this is the largest study of ICH predictors in adult patients on ECMO and contributes new findings that are important for patient management and future study design.

We included both VA and VV ECMO patients in our study, contrary to one of the previous studies that excluded VA patients on the basis of an increased risk of systemic thromboembolism from thrombus formation within the ECMO unit [15]. However, this complication is infrequent [24] due to the heparin infusion regimen as well as the bedside staffs’ continuous attentive observation of the ECMO circuit for signs of clotting. Moreover, to the best of our knowledge, a comparison between VA and VV ECMO of the frequency of systemic thromboembolism has not been studied in the adult population and is thus only theoretical, further supporting the inclusion of both patient categories in the analysis.

Twenty-one percent of our patients experienced an ICH during ECMO treatment. This is in the upper range of the 7–19% previously reported in similar studies [13–15]. However, in a number of cases, the ICHs we identified were diagnosed using CT scans performed in the absence of neurological symptoms (i.e., a cerebral CT scan that was performed at the same time as a CT scan of the thorax or abdomen). A previous study, conducted at our center, on adult and pediatric ECMO patients treated between 1994 and 2004 found that 24% of those with an intracranial pathology (defined as ICH, cerebral infarction, or general edema) presented with no clinical neurological signs before performing the diagnostic CT, further suggesting that low utilization of neuroimaging contributed to underreporting of ICH [25]. It is possible, therefore, that our CT examination policy was a contributing factor to the high diagnostic rate of ICH in our cohort. However, in previous studies, the routine for performing cerebral CT scans in the absence of neurological symptoms is poorly described, making comparison difficult. With respect to outcome, we found that the 1-month mortality rate for patients who experienced an ICH was 81%, compared to 28% in non-ICH patients, which is in accordance with current literature [8, 13–15]. Consequently, ICH was associated with a considerable risk for mortality in this ECMO population, with a somewhat higher incidence of ICH occurrence compared to previous studies.

Pulmonary indications were the most common reasons for ECMO treatment in this cohort (*n* = 224, 89%). Previously, other groups have tried to establish if the indication for ECMO was associated with a higher risk of ICH [13, 14] but were limited by few data points in several of the subgroups, making statistical analysis unreliable. Moreover, while we determined indications for ECMO treatment by denoting the most severely affected organ system, patients usually presented with failure of multiple organ systems, further confounding this parameter. Thus, we chose not to include this variable in further analysis.

Prior to our study, pre-admission antithrombotic therapy as a predictor of ICH had not been assessed in ECMO-treated adults [7, 13–15]. We identified the parameter as an independent predictor of ICH, with 50% of patients with pre-admission antithrombotic therapy who were admitted for ECMO treatment developing an ICH. In theory, long-term antithrombotic therapy could have rendered these patients hemostatically difficult to control using the heparin infusion regimen. Alternatively, there could be a prolonged drug effect at play that is difficult to take into account. However, we have not found any studies to guide us in this area. A limitation to the variable was that we did not differentiate between different forms of antithrombotics. This could be important as they have different mechanisms of action, although differentiating and subgrouping the antithrombotics would yield extremely small subgroups and make statistical modeling difficult. For descriptive purposes, a compilation of the different forms of pre-admission antithrombotic therapy is included in Additional file 2: Table S1. Our results imply that patients with pre-admission antithrombotic therapy were at increased risk of developing an ICH during ECMO treatment, but its specific role in different subgroups needs to be further evaluated.

We further identified low platelet count, amount of administered platelets, and high pre-cannulation SOFA coagulation score as predictors of ICH. Low platelet count, determined by the lowest platelet count recorded during the ECMO treatment, was identified as an independent predictor. Previously, Kasirajan et al. reported that thrombocytopenia was an independent predictor of ICH [13] but the results have not been confirmed by others, possibly due to a high platelet count and/or due to the low incidence of ICH in the patient cohorts in similar studies [14, 15]. The impact of high pre-cannulation SOFA coagulation score has been assessed once before, albeit not as a continuous variable, when Luyt et al. distinguished patients with a SOFA coagulation score >2, compared to those with a score ≤2, at ECMO initiation. However, in their cohort of 12 patients with an ICH, only one met these criteria [15]. The total amount of administered platelets was another predictor of ICH in our study and has been identified as such previously [14]. However, since patients are administered platelet transfusions to combat their low platelet count, thus introducing a potent treatment bias, this parameter can just as easily be interpreted as an identification of patients with thrombocytopenia rather than acting a risk factor on its own. Although this remains unknown, no prospective study has been designed to address the issue. Clinically, if there were no signs of bleeding, we used platelet counts <25–30 × 10^9^/mL as the arbitrary threshold for platelet transfusion. Lastly, in the temporal trajectories of patients with ICH, there was no significant change in platelet levels in the days leading up to the detection of an ICH. This can be attributed to the fact that not all bleedings were detected following the development of a neurological symptom(s), which makes it more difficult to determine the exact time of ictus. In addition, the values were achieved by calculating the daily average of each patients’ platelet count, which meant that momentary decreases, which could have led to hemorrhaging, did not show if the rest of the platelet counts that day were normal. Hence, low platelet count, both pre-cannulation and during ECMO treatment, was a predictor of ICH.

Spontaneous extracranial hemorrhage as a significant predictor of ICH in adult patients treated with ECMO has only been assessed once previously, with significant results [14]. This can be attributed to the loss of platelets and coagulation factors that occur following major bleeding, or to the fact that patients who are hemostatically unstable are more likely to bleed. The most common bleeding sites in our study were pulmonary, cannula insertion sites, gastrointestinal tract, abdominal cavity, and thoracic cavity. The amount of administered erythrocyte concentrate was also identified as a predictor of ICH. However, since patients are administered erythrocyte concentrate transfusions following major bleeding, this parameter is probably an identification of the patients with extracranial hemorrhage rather than a risk factor on its own, even if this remains unknown as no prospective study addressing this exists. Hence, spontaneous extracranial hemorrhage is associated with an increased risk for ICH but the precise mechanism behind it needs to be further assessed.

The risk of ICH was higher in patients who required dialysis, which is in accordance with previous studies; Kasirajan et al. showed that dialysis and hypercreatininemia predicted ICH development [13], and Luyt et al. showed a correlation between renal failure at ICU admission and increased risk of ICH [15]. However, the precise mechanism of action needs to be further assessed. Evidence is also warranted to determine if the increased risk of ICH is a result of the dialysis treatment or the conditions that require dialysis. Overhydration was the main indication for dialysis at our ECMO center, with acute kidney injury as the second most common indication, and the number of patients who received dialysis was high in both ICH and non-ICH cohorts (96 and 86%, respectively).

Finally, antithrombin significantly increased over time in patients who developed an ICH, with INR increase showing a trend towards significance. However, as shown in Fig. 1b, most ICH patients had antithrombin values within the reference range, making it difficult to draw conclusions based on the temporal trajectories alone. One could hypothesize that the significant temporal trajectory found was due to a general improvement of hemostatically unstable patients’ coagulation ability over time. Alternatively, it could be attributed to the fact that patients were, in some cases, administered antithrombin to combat heparin resistance, facilitate heparin effect, or reduce fibrin deposition in the ECMO circuit. However, the exact role of this correlation remains to be determined. Moreover, there was no significant change in APTT (Table 6), indicating that this per patient trajectory probably played a minor role in the development of ICH. The results also showed that the average patient had an APTT within the therapeutic range (Fig. 1a, Table 6). In the clinical setting, APTT is used to guide the heparin infusion regimen. This therefore suggests that the bleedings were not caused by heparin treatment errors (i.e., overdose). However, there are some solitary outliers in Fig. 1a, which could be the result of incorrect heparin administration or repetitive contamination when conducting the laboratory measurements of APTT. APTT was not included in the paired testing between ICH and non-ICH cohorts due to the risk of bias, as APPT is controlled by the amount of administered heparin and, accordingly, a non-therapeutic APTT also results in the physician adjusting the heparin infusion. To conclude, antithrombin significantly increased over time in patients who developed an ICH, but the clinical significance behind this is debatable. In addition, due to the lack of significant change in APTT in the days leading up to ICH diagnosis, coupled with the fact that the average patients’ APTT was within the therapeutic range, we concluded that the ICHs were not the result of incorrect heparin administration.

### Clinical implications and future research

Although this is a retrospective study, with its inherent limitations, our research highlights the importance of closely monitoring predictors of ICH. While we acknowledge that ECMO is a last resort treatment that is considered only after great scrutiny, it is important to identify the ICH predictors that can be targeted with interventions or where earlier weaning from ECMO could be attempted. Low platelet count can be combated by decreasing the threshold for platelet transfusion; this must however be balanced against the risk for thrombosis. Also, considering the fact that a number of patients developed ICH despite a normal mean platelet count, as is highlighted in Fig. 1e, one should consider the value of performing “thrombocyte function tests” (i.e., multiplate) on this patient group on a regular basis, as platelet dysfunction can lead to the development of ICH even in the absence of thrombocytopenia [26]. Pre-admission antithrombotic therapy, high pre-cannulation SOFA coagulation score, septic shock, dialysis, and spontaneous extracranial hemorrhage may help to identify the patients prone to ICH where more rigorous neurological checks and earlier weaning from ECMO could be attempted. Considering the high mortality associated with an ICH in patients on ECMO, prospective studies evaluating risk factors for ICH in this patient group are warranted, as well as studies on management and predictors of outcome after the occurrence of an ICH.

## Conclusions

ICH is a frequent complication in adult patients treated with ECMO and associated with increased mortality. We identified pre-admission antithrombotic therapy, low platelet count, high pre-cannulation SOFA coagulation score, spontaneous extracranial hemorrhage, dialysis, and septic shock as predictors of ICH during ECMO treatment, with pre-admission antithrombotic therapy and low platelet count identified as independent risk factors. In the clinical setting, risk factor identification may help initiate steps to lower the risk of ICH in patients undergoing ECMO treatment. Prospective trials are warranted to identify additional risk factors and their mechanisms of action.

## Additional files

Additional file 1: ECMO circuit. A description of the ECMO pumps, oxygenators, ventilators, cannulas and patients monitoring system used for the patients included in the study. (DOCX 84 kb)

Additional file 2: Table S1. A compilation of the different forms of pre-admission antithrombotic therapy in ICH vs. non-ICH cohorts. (DOCX 48 kb)

## Abbreviations

ACT: Activated clotting time
APTT: Activated partial thromboplastin time
CT: Computed tomography
ECMO: Extracorporeal membrane oxygenation
ECPR: Extracorporeal cardiopulmonary resuscitation
FiO_2_: Fraction of inspired oxygen
Fr: French
ICH: Intracranial hemorrhage
ICU: Intensive care unit
INR: International normalized ratio
MAP: Mean arterial blood pressure
P_a_O_2_: Arterial partial pressure of oxygen
P_v_CO_2_: Venous partial pressure of carbon dioxide
P_v_O_2_: Venous partial pressure of oxygen
S_cv_O_2_: Central venous oxygen saturation
SOFA: Sepsis-related organ failure assessment
UFH: Unfractionated heparin
VA ECMO: Venoarterial extracorporeal membrane oxygenation
VV ECMO: Venovenous extracorporeal membrane oxygenation
