# Correction to: Predictors of intracranial hemorrhage in adult patients on extracorporeal membrane oxygenation: an observational cohort study

**DOI:** 10.1186/s40560-019-0423-z

**Published:** 2020-01-03

**Authors:** Alexander Fletcher-Sandersjöö, Jiri Bartek, Eric Peter Thelin, Anders Eriksson, Adrian Elmi-Terander, Mikael Broman, Bo-Michael Bellander

**Affiliations:** 1 Department of Neurosurgery, Karolinska University Hospital, Stockholm, Sweden; 2 Department of Clinical Neuroscience, Karolinska Institutet, Stockholm, Sweden; 3 Department of Neurosurgery, Copenhagen University Hospital Rigshospitalet, Copenhagen, Denmark; 4 Division of Neurosurgery, Department of Clinical Neurosciences, Cambridge Biomedical Campus, University of Cambridge, Cambridge, UK; 5 ECMO Center Karolinska, Department of Pediatric Perioperative Medicine and Intensive Care, Karolinska University Hospital, Stockholm, Sweden; 6 Department of Physiology and Pharmacology, Karolinska Institutet, Stockholm, Sweden

**Correction to: J Intensive Care (2017) 5:27**

**https://doi.org/10.1186/s40560-017-0223-2**

In the original publication of this article [1], the first author’s name should be changed from Alexander Fletcher Sandersjöö to Alexander Fletcher-Sandersjöö. The family name of the author is Fletcher-Sandersjöö.

The authors sincerely apologize for the inconvenience caused to the readers.
