# Supplementary material for: Predictors of intracranial hemorrhage in adult patients on extracorporeal membrane oxygenation: an observational cohort study
Source: J Intensive Care. 2017 May 22;5:27. doi: 10.1186/s40560-017-0223-2 (PMC5441045; doi:10.1186/s40560-017-0223-2)
Supplement: Supplementary file 1 — ECMO circuit. A description of the ECMO pumps, oxygenators, ventilators, cannulas and patients monitoring system used for the patients included in the study. (DOCX 84 kb) [file 40560_2017_223_MOESM1_ESM.docx]

**ECMO circuit**

Biomedicus 550 consoles (Medtronic International Trading Sàrl, Tolochenaz, Switzerland) with a Rotaflow-pump (Maquet Cardiopulmonary AG, hirrlingen, Germany), Stöckert CAPS roller-pumps (Stöckert, Munich, Germany) or a Centrimag pump (Levitronix GmbH, Zurich, Switzerland) was used. The oxygenators employed were Medos Hilite 7000LT (Medos Medizintechnik AG, Stolberg, Germany) or Quadrox (Maquet). A Servo-I ventilator (Maquet Critical Care AB, Solna, Sweden) was used in the ICU, while a ResMed Elisee 250 (Maribo Medico A/S, Maribo, Denmark) or Hamilton T1-ventilator (Hamilton Medical AG, Bonaduz, Switzerland) was used during patient transport. All vascular catheterizations for ECMO were peripheral. The single-lumen cannulas used for VV or VA ECMO were Bio-Medicus (Medtronic) 15–21 French (Fr)/18 cm, 23 Fr/25 cm, 17–29 Fr/50 cm, or Maquet Venous hLS 25 Fr/38 cm. If dual lumen cannula technique was applied for VV ECMO, an OriGen 19 Fr, 23 Fr, 28 Fr (OriGen Biomedical GmbH, Burladingen, Germany), or an Avalon Elite 27 Fr, or 31 Fr (Maquet) was used. Blood-gases were assessed by an iSTAT System (Abbot Laboratories, Maidenhead, United Kingdom) and an IntelliVue X2 (Philips Healthcare, Best, Netherlands) was used for patient monitoring.
