# Supplementary material for: Predictors of intracranial hemorrhage in adult patients on extracorporeal membrane oxygenation: an observational cohort study
Source: J Intensive Care. 2017 May 22;5:27. doi: 10.1186/s40560-017-0223-2 (PMC5441045; doi:10.1186/s40560-017-0223-2)
Supplement: Supplementary file 2 — A compilation of the different forms of pre-admission antithrombotic therapy in ICH vs. non-ICH cohorts. (DOCX 48 kb) [file 40560_2017_223_MOESM2_ESM.docx]

**Additional file 2: Table S1**

**Supplementary table 1:** **ICH vs. non-ICH cohorts: Pre-admission antithrombotic therapy**

|  | **ICH cohort (n=54)** | **non-ICH cohort (n=199)** |
| --- | --- | --- |
| **Antithrombotic therapy** | **7 (13%)** | **7 (4%)** |
| Warfarin | 2 (4%) | 4 (2%) |
| ASA | 2 (4%) | 0 (0%) |
| ASA + Clopidogrel | 1 (2%) | 0 (0%) |
| ASA + Tinzaparin | 1 (2%) | 1 (1%) |
| Clopidogrel | 0 (0%) | 1 (1%) |
| Heparin | 1 (2%) | 0 (0%) |
| Dabigatran | 0 (0%) | 1 (1%) |

Abbreviations: ICH=Intracranial hemorrhage; ASA = Acetylsalicylic acid
